# Preparation of TiH_1.924_ nanodots by liquid-phase exfoliation for enhanced sonodynamic cancer therapy

**DOI:** 10.1038/s41467-020-17485-x

**Published:** 2020-07-24

**Authors:** Fei Gong, Liang Cheng, Nailin Yang, Yuehan Gong, Yanwen Ni, Shang Bai, Xianwen Wang, Muchao Chen, Qian Chen, Zhuang Liu

**Affiliations:** Institute of Functional Nano & Soft Materials (FUNSOM), Jiangsu Key Laboratory for Carbon-Based Functional Materials and Devices, Soochow University, Suzhou, 215123 China

**Keywords:** Cancer therapy, Nanoscale materials, Nanobiotechnology

## Abstract

Metal hydrides have been rarely used in biomedicine. Herein, we fabricate titanium hydride (TiH_1.924_) nanodots from its powder form via the liquid-phase exfoliation, and apply these metal hydride nanodots for effective cancer treatment. The liquid-phase exfoliation is an effective method to synthesize these metal hydride nanomaterials, and its efficiency is determined by the matching of surface energy between the solvent and the metal hydrides. The obtained TiH_1.924_ nanodots can produce reactive oxygen species (ROS) under ultrasound, presenting a highly efficient sono-sensitizing effect. Meanwhile, TiH_1.924_ nanodots with strong near-infrared (NIR) absorbance can serve as a robust photothermal agent. By using the mild photothermal effect to enhance intra-tumoral blood flow and improve tumor oxygenation, a remarkable synergistic therapeutic effect is achieved in the combined photothermal-sonodynamic therapy. Importantly, most of these TiH_1.924_ nanodots can be cleared out from the body. This work presents the promises of functional metal hydride nanomaterials for biomedical applications.

## Abstract (web summary)

Dynamic therapy is attracting attention for cancer treatment. Here, the authors report that metal hydride nanodots can be used for sonodynamic therapy, which can be further enhanced by photothermal heating to increase tissue oxygenation.

## Introduction

Sonodynamic therapy (SDT) triggered by ultrasound (US) is a non-invasive therapeutic strategy that can be applied to treat deeply-seated tumors^1–4^. During SDT, sono-sensitizers are able to interact with surrounding oxygen and even water molecules to produce cytotoxic reactive oxygen species (ROS) to kill tumor cells^2,4–7^. However, the limitations of current sono-sensitizers have substantially hindered the extensive clinical applications of SDT. Traditional organic sono-sensitizers (e.g., photofrin^8^, phthalocyanine^9^, and chlorophyll derivative^10^), which are often derived from photo-sensitizers, often show photo-toxicity toward the skin^11,12^. The most representative paradigm of inorganic sono-sensitizers is semiconductor titanium dioxide (TiO_2_)^13–15^, whose quantum yield of US-triggered ROS generation, however, is relatively low due to the fast combination of the electron (e^−^) and holes (h^+^) (50 ± 30 ns)^16,17^.

Titanium hydride (TiH_1.924_) has been explored for applications in hydrogen storage^18,19^, and is frequently used as a foaming agent in the production of metallic foams (e.g., Zn, Al foams)^20,21^, as well as a raw material for producing highly purified titanium and titanium alloys^22,23^. Considering the unique valence status of Ti (containing Ti^0^, Ti^2+^, Ti^3+^, and Ti^4+^) in TiH_1.924_,^24^ we hypothesize that it might be easily activated by external stimuli (e.g., light, ultrasound, and microwave) for applications in photo-catalysis and sono-catalysis^25,26^. However, nano-structured TiH_1.924_ has not yet been synthesized to our best knowledge.

**Fig. 1:**
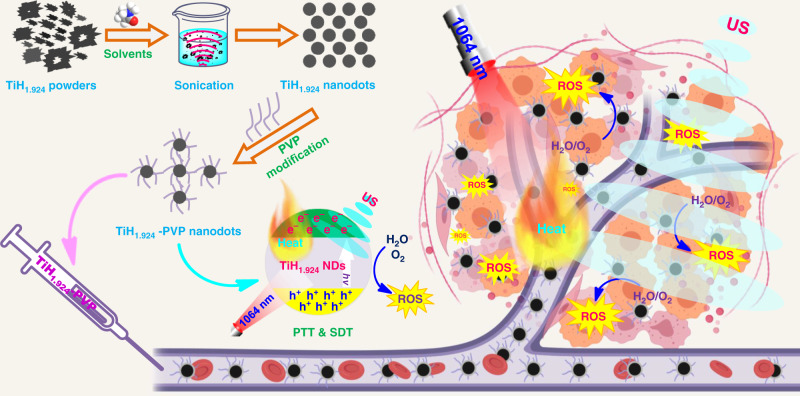
The preparation and application of TiH_1.924_ nanodot. Schematic illustration to show the preparation of TiH_1.924_ nanodots by liquid-phase exfoliation and their applications for combined photothermal-sonodynamic cancer therapy.

Liquid-phase exfoliation usually by sonicating bulk materials in appropriate solvents is a simple top-down route to produce various types of nanomaterials. In this work, we successfully exfoliate TiH_1.924_ powder into ultrasmall nanodots via the liquid-phase exfoliation technology, and find that the surface energy plays an important role in the formation of the ultrasmall TiH_1.924_ nanodots. In addition, this liquid-phase exfoliation is an effective method to synthesize various types of metal hydride nanomaterials (e.g., TiH_1.924_, ZrH_2_, CaH_2_, and HfH_1.983_). Taking TiH_1.924_ nanodots for example, they have highly effective US-triggered ROS generation capability, which is superior to the sono-sensitizing effect of titanium dioxide (TiO_2_), the classical inorganic sono-sensitizer, likely owing to the reduced bandgap in TiH_1.924_. Moreover, these black TiH_1.924_ nanodots with strong near-infrared (NIR) absorption can also use as an excellent photothermal agent. Taking the advantage of mild photothermal effect to enhance intra-tumor blood flow and improve oxygen supply, a remarkably synergistic photothermal-sonodynamic therapeutic outcome has been achieved with TiH_1.924_ nanodots (Fig. 1). In a mouse tumor model, the complete tumor eradication without recurrence is achieved after intravenous injection of TiH_1.924_ nanodots and exposure of tumors to light and ultrasound, sequentially. Importantly, these TiH_1.924_ nanodots with ultra-small sizes show efficient body excretion and no appreciable toxicity to the treated animals. This work highlights the potential of metal hydride nanomaterials as physical stimuli-triggered nanoagents for cancer treatment.

## Results

### Preparation and characterization of TiH_1.924_ nanodots

**Fig. 2:**
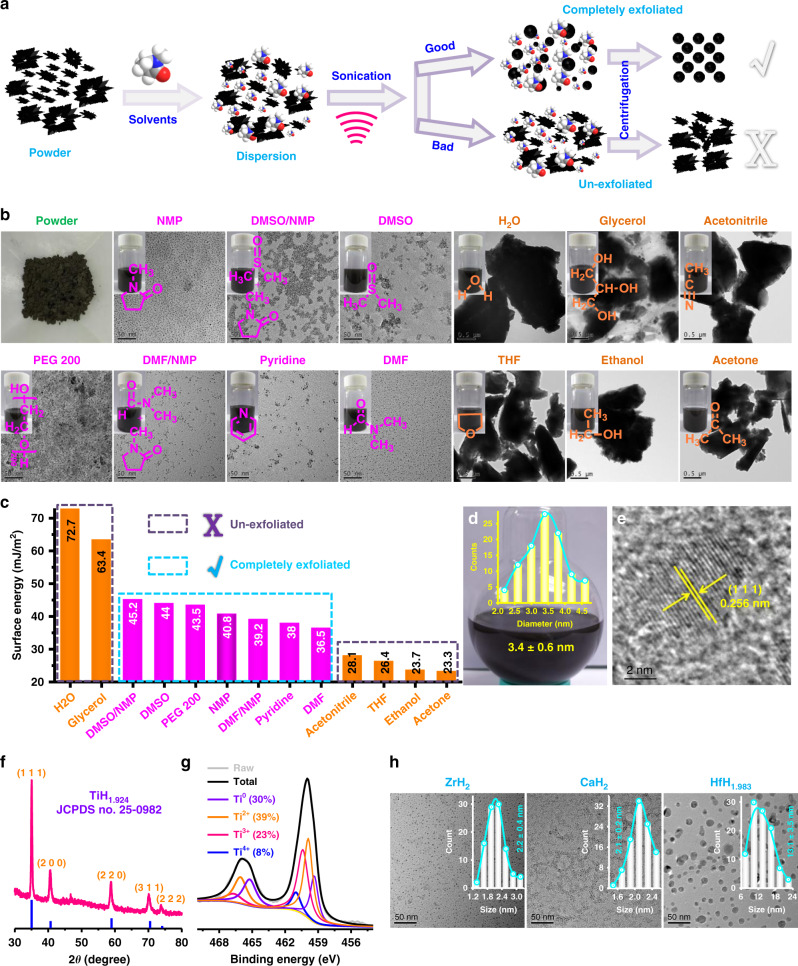
Preparation and characterization of TiH_1.924_ nanodots. **a** Schematic illustration to show light-phase exfoliation to prepare TiH_1.924_ nanodots. **b** A photograph of commercial TiH_1.924_ powder, the TEM images and corresponding photographs of exfoliated dispersions using various solvents (H_2_O, glycerol, dimethyl sulfoxide/*N*-methyl pyrrolidone (DMSO/NMP) DMSO/NMP, DMSO, polyethylene glycol 200 (PEG 200), NMP, *N,N*-dimethylformamide (DMF)/NMP, pyridine, DMF, acetonitrile, tetrahydrofuran (THF), ethanol, and acetone) for TiH_1.924_ exfoliation. **c** The surface energies of various solvents used to exfoliate TiH_1.924_. **d** A photograph of exfoliated TiH_1.924_ nanodots in NMP. Inset is the particle-size distribution (PSD) of TiH_1.924_ nanodots determined by the TEM image (*n* = 100 nanodots examined over TEM images). **e** High-resolution TEM (HRTEM) image of TiH_1.924_ nanodots. **f** XRD spectra of TiH_1.924_ nanodots. **g** XPS spectra to show Ti 2p peaks for the TiH_1.924_ nanodots sample. **h** TEM images and PSD of ZrH_2_ nanodots, CaH_2_ nanodots, and HfH_1.983_ nanoparticles exfoliated in NMP (*n* = 100 nanomaterials examined over TEM images). A representative image of three biological replicates from each group is shown in **b**, **e**.

Liquid-phase exfoliation technology has been widely reported for the preparation of mono- or few-layered two-dimensional nanosheets^27–30^. In this work, we unexpectedly found that metal hydrides powder could be easily exfoliated into nanodots using liquid-phase exfoliation technology in the presence of appropriate solvents (Fig. 2a). Taking TiH_1.924_ for example, we initially sonicated commercial TiH_1.924_ powder in a number of exfoliation solvents (Fig. 2a, b). Among these 13 solvents including water, glycerol, dimethyl sulfoxide/*N*-methyl pyrrolidone (DMSO/NMP), DMSO, polyethylene glycol 200 (PEG 200), NMP, *N,N*-dimethylformamide (DMF)/NMP, pyridine, DMF, acetonitrile, tetrahydrofuran (THF), ethanol and acetone, 6 solvents of them (water, glycerol, acetonitrile, THF, ethanol, and acetone) showed no exfoliation effects to the TiH_1.924_ powder, while the other 7 solvents (DMSO/NMP, DMSO, PEG 200, NMP, DMF/NMP, pyridine, and DMF) could efficiently exfoliate the TiH_1.924_ powder into small nanoparticles (Fig. 2b). The different exfoliation results might be attributed to the surface energy of these solvents (Fig. 2c)^27,31–33^. For these 13 solvents, when the surface energy is too high (H_2_O, 72.7; glycerol, 63.4 mJ m^−2^) or too low (acetonitrile, 28.1; THF, 26.3; ethanol, 23.7; acetone, 23.3 mJ m^−2^), TiH_1.924_ powder could not be effectively exfoliated. When the surface energy of the solvent (DMSO/NMP, 45.2; DMSO, 44; PEG 200, 43.5; NMP, 40.8; DMF/NMP, 39.2; pyridine, 38; DMF, 36.5 mJ m^−2^) reaches a range of 41 ± 5 mJ m^−2^, successful exfoliation of the TiH_1.924_ powder into small nanoparticles could be achieved. Therefore, we proposed that the successful exfoliation might be owing to the matching of surface energy between the applied solvents and the TiH_1.924_ powder.

Among these 13 solvents, NMP offered excellent exfoliation efficiency and the obtained TiH_1.924_ nanodots showed very uniform sizes and morphology (Fig. 2b). Thus, we employed NMP as the representative solvent to investigate the liquid-phase exfoliation of TiH_1.924_ powder. After exfoliation of TiH_1.924_ powder by sonication in NMP for different periods of time (Supplementary Fig. 1A–C), we found that the intensities of the X-ray diffraction (XRD) characteristic peaks decreased significantly by a time-dependent manner (Supplementary Fig. 1D). Particularly, after 20 min of ultrasonication, the TiH_1.924_ powder was entirely exfoliated into nanodots. Most importantly, this sample could be scaled-up to prepare large amounts of TiH_1.924_ nanodots with high quality, and the obtained TiH_1.924_ nanodots with an average diameter of 3.4 ± 0.6 nm could be well dispersed in NMP (Fig. 2d). The high-resolution TEM (transmission electron microscope) determined the lattice spacing to be 0.256 nm (Fig. 2e), which could be assigned to the (1 1 1) lattice plane of TiH_1.924_ (JCPDS No. 25-0982) (Fig. 2f)^34^. The energy dispersive spectrometer (EDS) spectrum also confirmed the existence of Ti elements (Supplementary Fig. 2). Based on the X-ray photoelectron spectroscopy (XPS, Supplementary Fig. 3), Ti with various valence states including Ti^0^ (30%), Ti^2+^ (39%), Ti^3+^ (23%), and Ti^4+^ (8%) were found in the obtained TiH_1.924_ nanodots (Fig. 2g)^24^. Apart from the TiH_1.924_ powder, we also successfully exfoliated zirconium hydride (ZrH_2_), calcium hydride (CaH_2_), and hafnium hydride (HfH_1.983_) powder using the liquid-phase exfoliation technology with the assistance of NMP (Fig. 2h, Supplementary Fig. 4). The obtained ZrH_2_ nanodots (2.2 ± 0.4 nm), CaH_2_ nanodots (2.1 ± 0.2 nm), and HfH_1.983_ nanoparticles (13.1 ± 3.5 nm) all showed uniform morphology, suggesting that the liquid-phase exfoliation technology could be a simple and universal method to prepare various types of metal hydrides nanomaterials.

### Sonodynamic and photothermal performance of TiH_1.924_ nanodots

**Fig. 3:**
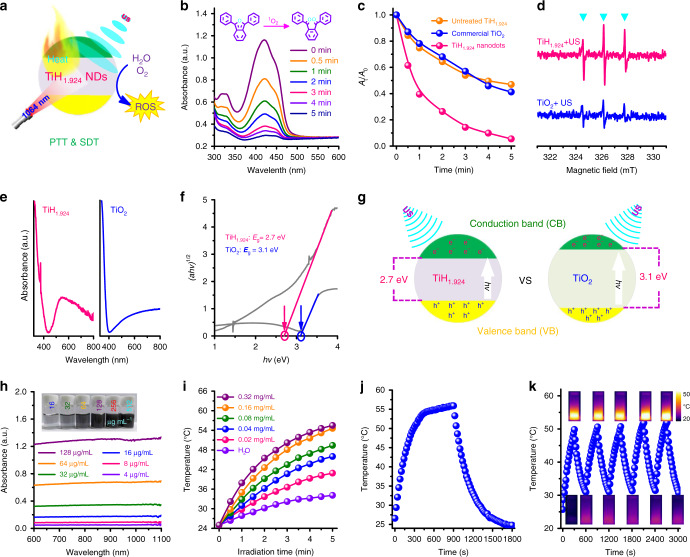
Sonodynamic and photothermal performance of TiH_1.924_ nanodots. **a** Schematic illustration of sonodynamic and photothermal properties of TiH_1.924_ nanodots. **b** Time-dependent oxidation of DPBF indicating ROS generation by US-activated TiH_1.924_ nanodots. **c** Comparison of DPBF oxidation by TiH_1.924_ nanodots, untreated TiH_1.924_, and commercial TiO_2_ under US irradiation for 5 min. **d** ESR spectra demonstrating ROS (^1^O_2_) generation for TiH_1.924_ and TiO_2_ under US irradiation for 1 min. **e**, **f** Normalized absorption spectra (**e**) and optical bandgaps (**f**) of TiH_1.924_ nanodots and TiO_2_. **g** Schematic illustration of the activation mechanism of TiH_1.924_ and TiO_2_ under US irradiation. **h** UV-vis-NIR absorbance spectra at different concentrations of TiH_1.924_ nanodots (4, 8, 16, 32, 64, and 128 µg mL^−1^). The inset is the photograph of TiH_1.924_ nanodots with different concentrations. **i** Concentration-dependent photothermal heating curves of TiH_1.924_ nanodots (0, 0.02, 0.04, 0.08, 0.16, and 0.32 mg mL^−1^). **j** The photothermal profile after laser exposure to reach a steady temperature and then to cool down by turning the laser off. **k** Heating/cooling profiles for five repeated ON-OFF cycles of laser irradiations.

The special valence structure of TiH_1.924_ nanodots indicates that they might be activated under US irradiation as a sono-sensitizer (Fig. 3a). Thus, to explore whether TiH_1.924_ nanodots could enhance sono-catalysis, 1,3-diphenylisobenzofuran (DPBF), a reactive oxide species (ROS) probe, was employed to detect the ROS generation by US-activated TiH_1.924_ nanodots. After mixing TiH_1.924_ nanodots with the DPBF probe, the UV-vis absorption spectrum of the mixture was monitored after different periods of ultrasound (US) irradiation (Fig. 3b, Supplementary Fig. 5). Undergoing a series of US irradiation time, the intensity of DPBF characteristic absorption peak at 420 nm showed significant decrease, suggesting the quenching of probe by the generated ROS. Compared with commercial TiO_2_ nanoparticles and untreated TiH_1.924_ powder, the exfoliated TiH_1.924_ nanodots exhibited a higher DPBF oxidation rate under the same US irradiation (Fig. 3c, Supplementary Fig. 6), indicating that TiH_1.924_ nanodots could serve as a stronger sono-sensitizer than TiO_2_. In addition, TiH_1.924_ sono-sensitizers with higher exfoliation degrees showed better sonodynamic performance (Supplementary Fig. 7). Electron spin resonance (ESR) detection was also performed to compare the generated ROS (^1^O_2_, ·O_2_^−^, and ·OH) between TiH_1.924_ and TiO_2_ sono-sensitizer. (Fig. 3d, Supplementary Figs. 8 and 9). The characteristic peak intensities of the TiH_1.924_ plus US showed a great increase than that of TiO_2_, further demonstrating that TiH_1.924_ nanodots could be activated to generate large amounts of ROS under US irradiation.

To understand the mechanism of sono-sensitization effect of TiH_1.924_ nanodots, the optical absorbance spectra of solid TiH_1.924_ nanodots and the commercial TiO_2_ nanoparticles were measured (Fig. 3e). Based on the optical absorbance spectra and the kubelka-munk theory (Fig. 3f)^35,36^, the optical bandgap of TiO_2_ was calculated to be ~3.1 eV, which was consistent with the previous reports^37–39^. Interestingly, the bandgap of TiH_1.924_ nanodots was determined to be ~2.7 eV, much lower than that of TiO_2_. The bandgap is related to the required minimum energy to realize electron excitation^40,41^. Thus, the lower bandgap means easier activation and would result in more ROS generation under external stimuli^42,43^. Based on the above discussion, the possible mechanism is proposed in Fig. 3g. Under the US irradiation, the valence electron receives energy and could transit from the valence band (VB) to the conduction band (CB), resulting in the generation of the electron-hole pairs and excess energy, which are captured by surrounding O_2_ and H_2_O molecules to generate ROS (e.g., ^1^O_2_, ·O_2_^−^, ·OH). With a lower bandgap compared to that of TiO_2_, TiH_1.924_ nanodots thus could be easier to be activated to produce more ROS under US irradiation, useful for applications in SDT.

We then studied the optical properties of the obtained TiH_1.924_ nanodots. The as-made TiH_1.924_ nanodots showed black color and strong optical absorbance, which appeared to be independent to the wavelength and was extended to the second NIR (NIR-II) region (Fig. 3h), in which light would have much higher tissue-penetrating capability in comparison to that in the NIR-I window^14,44^. On this ground, we presented that TiH_1.924_ nanodots could use as a photothermal agent for effective NIR-II PTT (1064 nm). The extinction coefficient of TiH_1.924_ at 1064 nm was tested to be ~10.27 L g^−1^ cm^−1^ (Supplementary Fig. 10), which was higher than that of black titania nanoparticles (B-TiO_2-*x*_, 5.54 L g^−1^ cm^−1^)^14^, traditional graphene oxide (GO, 3.6 L g^−1^ cm^−1^)^45^, and carbon nanodots (CQs, 0.35 L g^−1^ cm^−1^)^46^. Then, the photothermal performance of the TiH_1.924_ aqueous solution was further evaluated under 1064-nm NIR II laser. Significant concentration-dependent and laser-power-dependent photothermal heating effect was observed for these TiH_1.924_ nanodots (Fig. 3i, Supplementary Fig. 11). The photothermal conversion efficiency (*η*) of TiH_1.924_ nanodots was calculated to be ~58.6% (Fig. 3j, Supplementary Fig. 12), much higher than those of widely reported photothermal agents, like gold nanorods (21%)^47^, copper selenide (Cu_2-*x*_Se) nanocrystals (22%)^48^, copper sulfide (Cu_9_S_5_) nanocrystals (25.7%)^49^, and prussian blue (41.4%)^50^. In addition, there was almost no change of photothermal performance post five times ON/OFF laser cycles, showing the high photothermal stability of these TiH_1.924_ nanodots (Fig. 3k).

In order to increase their stabilities in the physiological environment, as-made TiH_1.924_ nanodots were modified with polyvinyl pyrrolidone (PVP), which could stabilize TiH_1.924_ nanodots likely via the chelating-coordination between the O atoms of PVP and Ti atoms of TiH_1.924_ (Supplementary Fig. 13)^44,51^. The amount of PVP coated on the surface of TiH_1.924_ nanodots was measured by thermogravimetric analysis (TGA) to be ~26.4%. Unlike as-made TiH_1.924_ nanodots which could be well dispersed in water but would aggregate in the presence of salt (e.g., in phosphate-buffered saline, PBS), TiH_1.924_-PVP nanodots showed great dispersity in both water, PBS, and cell culture medium for a week. Notably, the photothermal and sonodynamic performance of TiH_1.924_ nanodots did not change after surface modification with PVP or additional H_2_O_2_ treatments (Supplementary Figs. 14–16).

### In vitro mild PTT-enhanced SDT

**Fig. 4:**
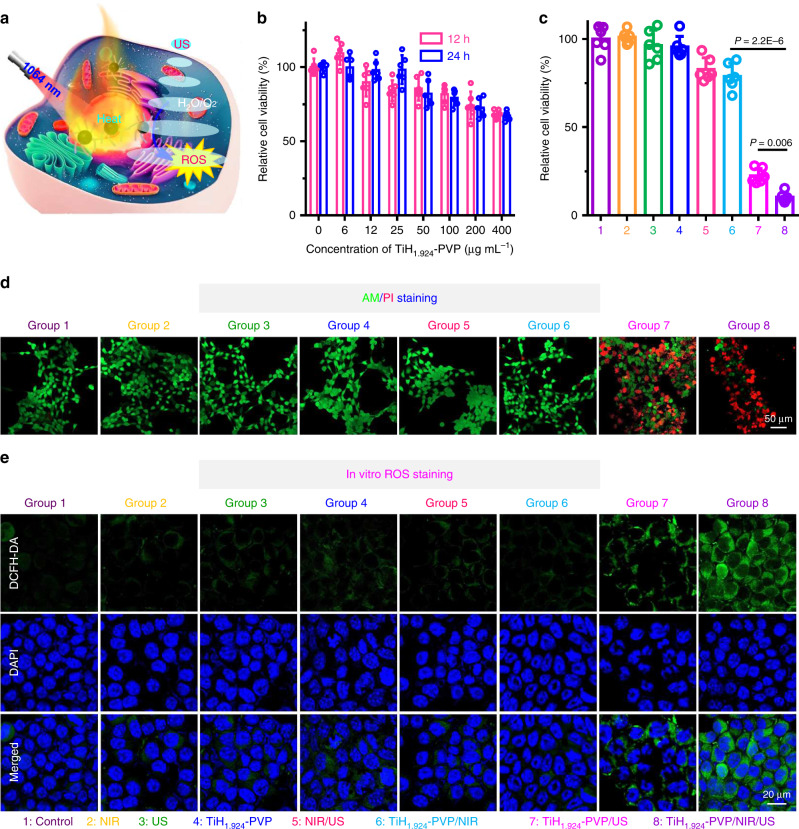
In vitro mild PTT-enhanced SDT via TiH_1.924_-PVP. **a** Schematic illustration of TiH_1.924_-PVP for mild PTT-enhanced sonodynamic therapy. **b** Relative viabilities of 4T1 cells after incubation with various concentrations of TiH_1.924_-PVP for 12 h and 24 h (*n* = 6 biologically independent samples). **c** Relative viabilities of 4T1 cells after different treatments, including control, TiH_1.924_-PVP, NIR, US, NIR/US, TiH_1.924_-PVP/NIR, TiH_1.924_-PVP/US, and TiH_1.924_-PVP/NIR/US (*n* = 6 biologically independent samples). **d** Confocal images of 4T1 cells stained with calcein AM (green, live cells) and propidium iodide (red, dead cells) after different treatments. **e** Confocal images of 4T1 cells stained with DCFH-DA after various treatments. The nuclei and intracellular ROS were stained by DAPI (blue) and DCFH-DA (green), respectively. TiH_1.924_-PVP: 50 µg mL^−1^, NIR laser: 1064 nm, 0.8 W cm^−2^, 10 min, *T* < 42 °C; US irradiation: 40 kHz, 3 W cm^−2^, 1 min per cycle, 5 cycles. Data are presented as mean values ± SD. Statistical significance was calculated with two-tailed Student’s *t* test (**c**). A representative image of three biological replicates from each group is shown in **d**, **e**.

With strong NIR-II absorbance and effective sono-sensitizing ability, we expected the utilization of TiH_1.924_-PVP for synergistic photothermal-sonodynamic cancer therapy (Fig. 4a, Supplementary Fig. 17). Firstly, the standard methyl thiazolyl tetrazolium (MTT) assay demonstrated that TiH_1.924_-PVP nanodots show negligible cytotoxicity even at high concentrations (400 µg mL^−1^) toward 4T1 tumor cells (Fig. 4b). Next, the in vitro PTT-enhanced SDT induced by TiH_1.924_-PVP was evaluated (Fig. 4c). After the mild PTT using the 1064-nm laser at the power density of 0.8 W cm^−2^ for 10 min, the cell culture temperature increased to ~42 °C and the cell viability incubated with TiH_1.924_-PVP showed a slight decrease (~80.9%). When further US irradiation was conducted (40 kHz, 3 W cm^−2^, 1 min per cycle, 5 cycles), the 4T1 cell viabilities significantly decreased to ~10.6%, presenting increased cell killing compared to TiH_1.924_-PVP treated cells exposed to US alone without pre-treatment by the NIR-II laser. In addition, the excellent cancer cell killing effect of mild PTT-enhanced SDT using TiH_1.924_-PVP was also confirmed by live/dead co-staining (live cells, calcein-AM, AM; dead cells, propidium iodide, PI) (Fig. 4d). This increased SDT performance may be ascribed to the mechanism that the laser treatment could change the cell membrane permeability and enhance the cell uptake of TiH_1.924_-PVP nanodots^52,53^.

Next, 2,7-dichlorofluorescein diacetate (DCFH-DA, green color) and dihydroethidium (DHE, red color) staining assays were also performed to determine intracellular ROS generation and verify the mechanism of TiH_1.924_-PVP as a sono-sensitizer to kill cancer cells under ultrasound (Fig. 4e, Supplementary Figs. 18 and 19)^54^. Cells in the control group, TiH_1.924_-PVP only group, laser only group, US only group, laser/US group, and TiH_1.924_-PVP/NIR group (mild PTT), all showed weak intracellular ROS-related fluorescence. In contrast, strong fluorescent signals were clearly observed in cells from the TiH_1.924_-PVP/US and TiH_1.924_-PVP/NIR/US groups, demonstrating the effective intracellular ROS generation by TiH_1.924_-PVP under US stimulation.

### Mild PTT-defeated tumor hypoxia

**Fig. 5:**
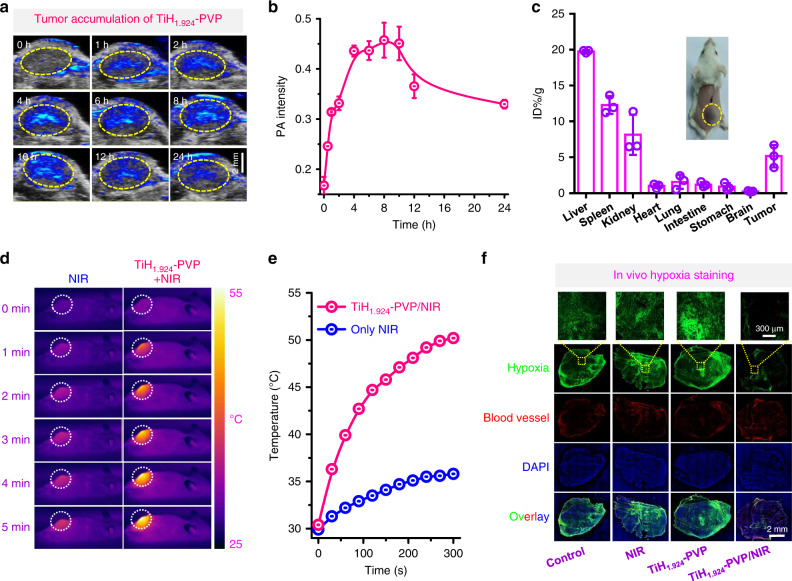
In vivo tumor accumulation and mild PTT-defeated tumor hypoxia via TiH_1.924_-PVP. **a** In vivo PA imaging of 4T1 tumor-bearing mice after intravenously injected with TiH_1.924_-PVP. **b** Time-dependent tumor PA signals at 900 nm based on PA imaging data in **a** (*n* = 3 biologically independent mice). **c** Biodistribution of TiH_1.924_-PVP in mice (*n* = 3 biologically independent mice). **d**, **e** IR thermal images (**d**) and temperature change curves (**e**) of 4T1 tumors under the 1064-nm laser irradiation, for untreated mice and TiH_1.924_-PVP injected mice (irradiated at 8 h p.i.). **f** Representative immunofluorescence images of tumor slices after hypoxia staining. The nuclei, blood vessels, and hypoxia areas were stained by DAPI (blue), anti-CD31 antibody (red), and antipimonidazole antibody (green), respectively. TiH_1.924_-PVP: 20 mg kg^−1^; NIR laser: 1064 nm, 0.8 W cm^−2^, 20 min. A representative image of three biological replicates from each group is shown in **f**. Data are presented as mean values ± SD.

After in vitro experiments, the in vivo behaviors of TiH_1.924_-PVP were studied using photoacoustic (PA) imaging and it could monitor the tumor uptake of NIR-absorbing TiH_1.924_-PVP nanodots. After intravenous (i.v.) injection of TiH_1.924_-PVP into 4T1-tumor-bearing balb/c mice for 8 h, much obvious PA signals were clearly appeared in the tumor site (Fig. 5a, b), verified the tumor uptake of TiH_1.924_-PVP via the enhanced permeability and retention (EPR) effect. At the following time points, the PA signals gradually decrease, likely due to the clearance of the ultrasmall TiH_1.924_-PVP from the tumor. In addition, the biodistribution of nanodots in the tumor was then quantitatively studied by measuring the content of titanium ions through inductively coupled plasma optical emission spectrometry (ICP-OES) at 8 h post injection (p.i.). The tumor uptake of TiH_1.924_-PVP was determined to be ~5.2%ID g^−1^, further confirming the efficient tumor accumulation of these nanodots (Fig. 5c).

Afterward, the in vivo photothermal performance of TiH_1.924_-PVP for NIR II-induced hyperthermia was investigated own to the strong NIR absorbance and high tumor accumulation of TiH_1.924_-PVP nanodots. And the surface temperature of tumors was recorded by infrared (IR) thermal imaging. 4T1 tumors-bearing mice post i.v. injection of TiH_1.924_-PVP for 8 h were exposed to the 1064-nm laser irradiation (0.8 W cm^−2^, 5 min) (Fig. 5d, e). Obviously, the tumor temperatures for TiH_1.924_-PVP treated mice quickly increased to 50 °C, while that of the control group showed much less significant temperature increase.

Due to aberrant cancer cell proliferation and distorted blood tumor vessels, hypoxia arises in a wide variety of solid tumors and often causes the failure of cancer therapies, especially for those that consume oxygen in the cell killing process such as radiotherapy, photodynamic therapy (PDT), and SDT^55–57^. Based on previous reports, the mild photothermal effect may increase intra-tumoral blood flow and then overcome tumor hypoxia^58–60^. To confirm this effect, immune-fluorescence hypoxia staining assay was conducted (Fig. 5f). Obviously, TiH_1.924_-PVP plus NIR-II irradiation group showed a significantly decrease of the hypoxia signals, suggesting that the mild photothermal effect could efficiently overcome the tumor hypoxia, favorable for defeating hypoxia-associated SDT resistance.

### In vivo mild PTT-enhanced SDT

**Fig. 6:**
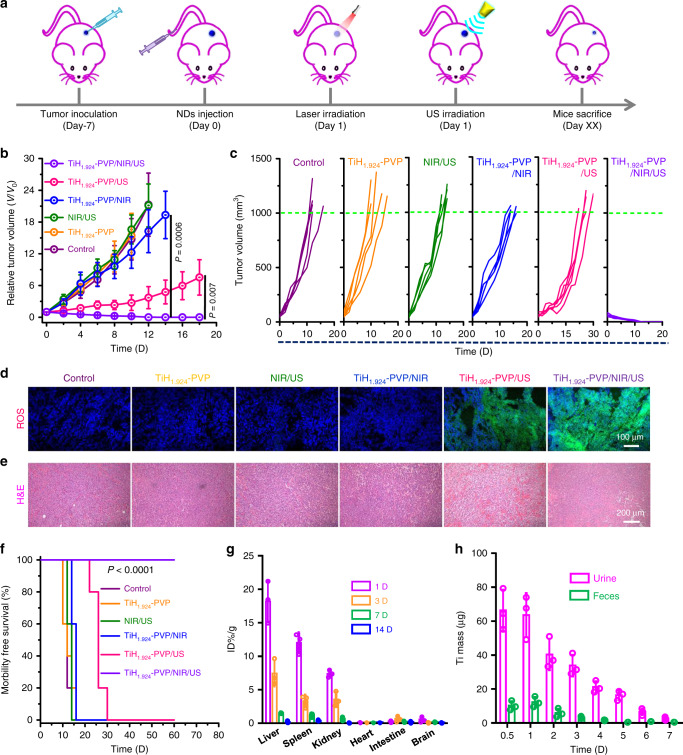
In vivo mild PTT-enhanced SDT via TiH_1.924_-PVP. **a** Schematic illustration to show the combination of PTT and SDT with TiH_1.924_-PVP nanodots. **b**, **c** Average tumor growth curves (**b**) and individual tumor growth curves (**c**) on mice after different treatments, including control, TiH_1.924_-PVP, NIR/US, TiH_1.924_-PVP/NIR, TiH_1.924_-PVP/US, and TiH_1.924_-PVP/NIR/US (*n* = 5 biologically independent mice). **d** Micrograph of DCFH-DA stained tumor slices collected for mice receiving different treatments. **e** H&E stained tumor slices collected from different treatment groups. **f** Survival curves of mice after various treatments. **g** Biodistribution of TiH_1.924_-PVP post i.v. injection in mice on different days (*n* = 3 biologically independent mice). **h** The detected Ti mass in urine and feces at different time points post i.v. injection of TiH_1.924_-PVP (*n* = 3 biologically independent mice). The Ti contents were measured by ICP-OES. TiH_1.924_-PVP: 20 mg kg^−1^; NIR laser: 1064 nm, 0.8 W cm^−2^, 20 min, *T* < 45 °C; US irradiation: 40 kHz, 3 W cm^−2^, 1 min per cycle, 20 cycles. A representative image of three biological replicates from each group is shown in **d**, **e**. Data are presented as mean values ± SD. Statistical significance was calculated with two-tailed Student’s *t* test (**b**) and Logrank test (two-sided) for trend (**f**).

Then, we conducted the mild PTT-enhanced SDT on 4T1 tumor-bearing mice using TiH_1.924_-PVP. All of the mice were divided into five groups: (1) Control; (2) TiH_1.924_-PVP (i.v. injection, 20 mg kg^−1^); (3) NIR (1064 nm, 0.8 W cm^−2^, 20 min, *T* < 45 °C) + US (40 kHz, 3 W cm^−2^, 1 min per cycle, 20 cycles); (4) TiH_1.924_-PVP + NIR; (5) TiH_1.924_-PVP + US; (6) TiH_1.924_-PVP + NIR + US. At 8 h post i.v. injection of TiH_1.924_-PVP, the tumors were treated with 1064 nm laser and subsequent US irradiation (Fig. 6a). After various treatments, the tumor growth on different groups of mice was monitored. Compared with control, TiH_1.924_-PVP injection alone, or NIR/US treated for saline injected mice showed no appreciable effect to the tumor group (Fig. 6b, c, Supplementary Fig. 20A). The mild PTT with TiH_1.924_-PVP could only partially inhibit the tumor growth. The tumor growth of the SDT group (TiH_1.924_-PVP/US) was remarkably suppressed, suggesting the excellent SDT performance of TiH_1.924_-PVP. Interestingly, the TiH_1.924_-PVP/NIR/US group showed the most remarkable therapeutic outcome, and the tumor tissues were completely eradiated without recurrence during two months. The survival time of mice in the SDT group (TiH_1.924_-PVP/US) was prolonged compared to the other four groups (control, TiH_1.924_-PVP, NIR/US, and mild PTT group) (Fig. 6f). More importantly, the mice in the mild PTT-enhanced SDT group showed 100% survival for two months, further demonstrating an obvious synergistic therapeutic outcome for the combined PTT-SDT with TiH_1.924_-PVP in comparison to the single-modality SDT or mild PTT.

To further understand the mechanism of synergistic therapy, ROS staining of tumor slices was conducted to evaluate the ROS levels in the tumor post different treatments (Fig. 6d). Compared with the weak green fluorescence in tumor slices from control, TiH_1.924_-PVP, NIR/US, and TiH_1.924_-PVP/NIR groups, the TiH_1.924_-PVP/US group showed obvious green fluorescence, and the strongest ROS-related fluorescence was observed in the combined laser plus US treatment group (TiH_1.924_-PVP/NIR/US). Our results indicated the mild PTT could overcome tumor hypoxia and facilitate the SDT-triggered ROS production. In addition, hematoxylin and eosin (H&E) staining were conducted at 24 h after different treatments. Tumor cells were severely damaged in the SDT group and mild PTT-enhanced SDT group, while the other four groups showed little cell dead (Fig. 6e). These results confirmed the efficient synergistic effects induced by mild PTT-enhanced SDT, in the presence of TiH_1.924_-PVP as a concurrent sono-sensitizer and photothermal nanoagent.

### The body clearance behaviors

In two weeks after the treatment, the body weights of mice showed no significant change, indicating no apparent acute toxicity of TiH_1.924_-PVP (Supplementary Fig. 20B). Next, we further investigated the body clearance behaviors of TiH_1.924_-PVP after systemic injection, and a time-dependent biodistribution study was conducted after i.v. injection of TiH_1.924_-PVP nanodots (Fig. 6g). Relatively high retention of TiH_1.924_-PVP was observed in the liver (18.2 ± 3.1%ID g^−1^), spleen (12.1 ± 1.5%ID g^−1^), and kidney (7.4 ± 0.5%ID g^−1^) at 24 h p.i. Importantly, rapid decrease of Ti levels in these organs was observed over time, indicating the efficient clearance of TiH_1.924_-PVP. After 14 days, the Ti retention in major organs drastically decreased to be <0.5%ID g^−1^, indicating the nearly complete clearance of TiH_1.924_-PVP. To further investigate the clearance pathway, the Ti concentrations in the urine and feces were also measured. High levels of Ti were observed in the urine, strongly evidencing the elimination of TiH_1.924_-PVP nanodots via the renal filtration pathway (Fig. 6h). In addition, H&E staining of the major organs (heart, liver, spleen, kidney, heart, lung, and brain) also confirmed the negligible toxicity of TiH_1.924_-PVP to mice at this therapeutic dose (Supplementary Fig. 21). With efficient clearance and no acute toxicity, such TiH_1.924_-PVP nanodots could be safe for in vivo use without long-term toxicity within the appropriate dose range.

## Discussion

In summary, nano-structured TiH_1.924_ materials were synthesized via the liquid-phase exfoliation method. It was found that when the surface energy of the applied solvent had a good match with that of the TiH_1.924_ powder, efficient exfoliation of such metal hydride powder into nanoparticles could be realized. Using the same method, a series of metal hydrides powders (TiH_1.924_, ZrH_2_, CaH_2_, and HfH_1.983_) were successfully exfoliated into small nanoparticles. With strong NIR-II absorbance and efficient US-triggered ROS production ability, TiH_1.924_ nanodots with PVP modification were further applied for the combined PTT-SDT therapy with great in vivo tumor destruction efficacy. Such TiH_1.924_ nanodots present the following advantages as therapeutic nano-agent. (1) These TiH_1.924_-PVP nanodots exhibit excellent sonodynamic performance in US-triggered ROS generation due to the reduced band-gap in TiH_1.924_ compared to that of TiO_2_. (2) The strong NIR absorption of TiH_1.924_-PVP could enable enhanced photothermal-sonodynamic therapy, in which the mild hyperthermia-induced tumor hypoxia relief would lead to improved sonodynamic tumor killing. (3) Containing biocompatible elements (Ti and H), TiH_1.924_-PVP nanodots with ultra-small sizes could allow their efficient body excretion without appreciable toxicity. Moreover, this work illustrates the promises of nano-structured metal hydrides nanomedicine platform against cancer and possibly other types of diseases.

## Methods

### Materials

Titanium hydride (TiH_1.924_), zirconium hydride (ZrH_2_), calcium hydride (CaH_2_), hafnium hydride (HfH_1.983_), and *N*-methyl pyrrolidone (NMP) were purchased from Aladdin reagent Co., Ltd. (Shanghai, China). 1,3-diphenylisobenzofuran (DPBF), Polyvinyl pyrrolidone (PVP, MW 10 k), 2,2,6,6-tetramethylpiperidine (TEMP), and 5,5-dimethyl-pyrroline-*N*-oxide (DMPO) were obtained from Sigma-Aldrich. All chemicals were of analytical grade and used without further purification.

### Synthesis of TiH_1.924_ nanodots

100 mg commercial TiH_1.924_ powder was dispersed in 20 mL NMP. The mixture was treated under ultrasonication for different periods of time (Ultrasonic Cleaner, KQ-100KDB, power: ~100 W, temperature: ~15 °C). After ultrasonic treatment for 20 min, TiH_1.924_ nanodots were obtained and further purified by centrifugation (64 k × *g*, 10 min) and washing repeatedly with anhydrous ethanol. Via the same method, zirconium hydride (ZrH_2_) nanodots, calcium hydride (CaH_2_) nanodots, and hafnium hydride (HfH_1.983_) nanoparticles were also synthesized.

### Modification of TiH_1.924_ nanodots

The as-synthesized TiH_1.924_ nanodots were modified by polyvinylpyrrolidone (PVP)^44,51^. Briefly, 20 mg TiH_1.924_ and 200 mg PVP (MW 10 k) were dissolved in 50 mL anhydrous ethanol and refluxed at 50 °C for 8 h. After collecting by centrifugation (64 k × *g*, 10 min) and washing with water and ethanol, the final TiH_1.924_-PVP nanodots were dispersed in deionized water, and stored at 4 °C for further use (concentration, 2 mg mL^−1^).

### Characterization

Transmission electron microscope (TEM) imaging and elemental mapping were carried out by FEI Tecnai F20 TEM. Powder X-ray diffraction (XRD) measurement was conducted by a PANalytical X-ray diffractometer equipped with CuKα radiation (*λ* = 0.15406 nm). XPS analysis was performed by the PHI Quantera SXM X-ray photoelectron spectrometer with an Al Ka monochromator source. ROS was quantified by an ESR spectrometer (Bruker EMXplus). UV-vis-NIR absorbance spectra were recorded by PerkinElmer Lambda 750 UV-vis-NIR spectrophotometer. The ultrasonic generator was made by Hainertec (Suzhou) Co., Ltd. The absolute Ti contents were determined by ICP-OES (inductively coupled plasma optical emission spectrometry).

### Quantitative analysis of the generation of ROS

1 mL TiH_1.924_ (20 µg mL^−1^) was mixed with 20 μL DPBF (1 mg mL^−1^). After different US (40 kHz, 3 W cm^−2^) durations, the absorbance changes of DPBF at 420 nm were recorded to quantify the generation of ROS by US-activated TiH_1.924_. ESR technology combined with TEMP (for ^1^O_2_ detection) or DMPO (for ·OH detection) was employed to detect different types of the generated ROS. In this case, 1 mL TiH_1.924_ (20 µg mL^−1^) was mixed with 20 µL TEMP (1 M) or 10 µL DMPO (1 M) and exposed to US irradiation (40 kHz, 3 W cm^−2^) for 1 min. The characteristic peak signals were detected by the ESR spectrometer. The settings for the EPR spectrometer were as follows: center field, 3520 G; sweep width, 100 G; microwave frequency, 9.77 GHz; modulation frequency, 100 kHz; power, 20.00 mW.

### Photothermal performance of TiH_1.924_ nanodots

The photothermal performance of TiH_1.924_ was analyzed by irradiating a glass cuvette containing a dispersion of TiH_1.924_ nanodots. The extinction coefficient and the photothermal conversion efficiency were calculated according to the previous studies^44^.

### Cellular experiments

Murine breast cancer 4T1 cells were cultured in the standard cell culture medium at 37 °C under 5% CO_2_. For the in vitro cytotoxicity test, 4T1 cells seeded in 96-well plates were incubated with different concentrations (0-400 µg mL^−1^) of TiH_1.924_-PVP for 12 h and 24 h. Relative cell viabilities were tested by the standard MTT assay.

For in vitro mild PTT-enhanced SDT, 4T1 cells were incubated with TiH_1.924_-PVP (50 µg·mL^−1^) for 8 h, followed by laser irradiation (1064 nm, 0.8 W cm^−2^, 10 min, *T* < 42 °C) or US irradiation (40 kHz, 3 W cm^−2^, 1 min per cycle, 5 cycles). The cell viabilities were determined afterward by the MTT assay.

For live/dead staining, 4T1 cells under different treatments (including control, TiH_1.924_-PVP, NIR, US, NIR/US, TiH_1.924_-PVP/NIR, TiH_1.924_-PVP/US and TiH_1.924_-PVP/NIR/US) were stained with calcein AM (AM, live cell) and propidium iodide (PI, dead cell). For ROS detection, the treated 4T1 cells were stained with DCFH-DA (20 μM) for 30 min. All the images were acquired by a confocal laser scanning microscope (CLSM, Zeiss Axio-Imager LSM-800).

### Tumor model

Balb/c mice were purchased from Nanjing Sikerui Biological Technology Co. Ltd, and all animal experiments were carried out under the permission by Soochow University Laboratory Animal Center. Six-week-old male Balb/c mice (18 ± 2 g) were used as the animal model in this work. Mice were housed in groups of 5 mice per individually ventilated cage in a 12-h light–dark cycle (8:00–20:00 light; 20:00–8:00 dark), with constant room temperature (21 ± 1 °C) and relative humidity (40-70%). All mice had access to food and water ad libitum.

### Hypoxia tumor analysis

For immunohistochemistry analysis, 4T1 tumor-bearing mice were intravenously injected with TiH_1.924_-PVP (20 mg·kg^−1^). At 8 h p.i., tumors on these mice were exposed to the 1064-nm laser irradiation for 20 min with their temperature maintained at ~45 °C. Then immediately, tumors were surgically excised for hypoxia staining assay using the Hypoxyprobe-1 plus kit (Hypoxyprobe Inc) following the standard protocol^61,62^. Anti-pimonidazole mouse monoclonal antibody conjugated with FITC (FITC-Mab1, Hypoxyprobe Inc.; Cat. No.: HP2-100Kit; Lot No.: 04-11-19; Clone: 4.3.11.3; Dilution: 1:200) and Alex 488-conjugated goat anti-mouse secondary antibody (Jackon Inc., Cat. No.: 115-545-003, Lot No.: 146108, RRID: AB_2338840; dilution: 1:200) for hypoxia staining. Rat anti-CD31 mouse monoclonal antibody (Biolegend Inc., Cat. No.: 102402, Lot No.: B226360, Clone: 390; dilution: 1:100) and Rhodamine-conjugated donkey anti-rat secondary antibody (Jackon Inc. Cat. No.: 712-025-150, Lot No.: 147079, RRID: AB_2340635; Dilution: 1:200) for blood vessel staining.

### In vivo mild PTT enhanced SDT

Mice bearing 4T1 tumors (~100 cm^3^) were divided into six groups (*n* = 5 per group): (1) control; (2) TiH_1.924_-PVP (i.v. injection, 20 mg kg^−1^); (3) NIR (1064 nm, 0.8 W cm^−2^, 20 min, *T* < 45 °C) + US (40 kHz, 3 W cm^−2^, 1 min per cycle, 20 cycles); (4) TiH_1.924_-PVP (i.v. injection, 20 mg kg^−1^) + NIR (1064 nm, 0.8 W cm^−2^, 20 min, *T* < 45 °C); (5) TiH_1.924_-PVP (i.v. injection, 20 mg kg^−1^) + US (40 kHz, 3 W cm^−2^, 1 min per cycle, 20 cycles); (6) TiH_1.924_-PVP (i.v. injection, 20 mg kg^−1^) + NIR (1064 nm, 0.8 W cm^−2^, 20 min, *T* < 45 °C) + US (40 kHz, 3 W cm^−2^, 1 min per cycle, 20 cycles). At 8 h after i.v. injection, the tumors were treated with laser irradiation, or US irradiation, or laser irradiation + US exposure, sequentially. Tumor temperature and thermal images were monitored and recorded by an IR thermal camera (Infrared Cameras. Inc). Tumor sizes and body weight were monitored every two days. The tumor volumes were calculated by the formula: volume = length × width^2^/2. For in vivo H&E staining, tumors in different groups were collected on the second day post treatment.

### In vivo metabolism study

Healthy mice after i.v. injection with TiH_1.924_-PVP (20 mg·kg^−1^) was sacrificed at 1, 3, 7, and 14 days, respectively. The major organs were collected, with one halves used for H&E staining, and the other halves used for detection of Ti levels by ICP-OES after these organs were solubilized by aqua regia. To study the excretion pathway, mice after i.v. injection with TiH_1.924_-PVP nanodots were kept in metabolic cages to collect their feces and urine at various time points, which were solubilized by aqua regia measured by ICP-OES to determine Ti levels.

### Software

All statistical analyses were performed on Origin 8.5, Excel 2010 and GraphPad Prism 6. Fluorescent images were collected by Confocal Microscopy (Zeiss LSM 880) and analyzed by LAS AF Lite 3.2.0 Image J 1.74v. IR thermal images were collected by Infrared Camera (Fotric 255). Photoacoustic imaging data was processed by PA Tomography (Vevo LAZR). All other characterization of TiH_1.924_ was conducted by these instruments as indicated in the Characterization section.

### Reporting summary

Further information on research design is available in the Nature Research Reporting Summary linked to this article.

## Supplementary information

Supplementary Information

Peer Review File

Reporting Summary

## Data Availability

The authors declare that all data needed to evaluate the conclusion of this work are presented in the paper and the Supplementary Information. Other data related to this work are available from the corresponding authors upon reasonable request.
