# Supplementary material for: Preparation of TiH1.924 nanodots by liquid-phase exfoliation for enhanced sonodynamic cancer therapy
Source: Nat Commun. 2020 Jul 24;11:3712. doi: 10.1038/s41467-020-17485-x (PMC7381661; doi:10.1038/s41467-020-17485-x)
Supplement: Supplementary file 1 — Supplementary Information [file 41467_2020_17485_MOESM1_ESM.pdf]

Supplementary Information

Preparation of $TiH_{1.924}$ Nanodots by Liquid-phase Exfoliation for Enhanced Sonodynamic Cancer Therapy

Fei Gong, Liang Cheng*, Nailin Yang, Yuehan Gong, Yanwen Ni, Shang Bai, Xianwen Wang, Muchao Chen, Qian Chen, Zhuang Liu*

Institute of Functional Nano & Soft Materials (FUNSOM), Jiangsu Key Laboratory for Carbon-Based Functional Materials and Devices, Soochow University, Suzhou 215123, China.

*E-mail: lcheng2@suda.edu.cn (L. Cheng), E-mail: zliu@suda.edu.cn (Z. Liu)

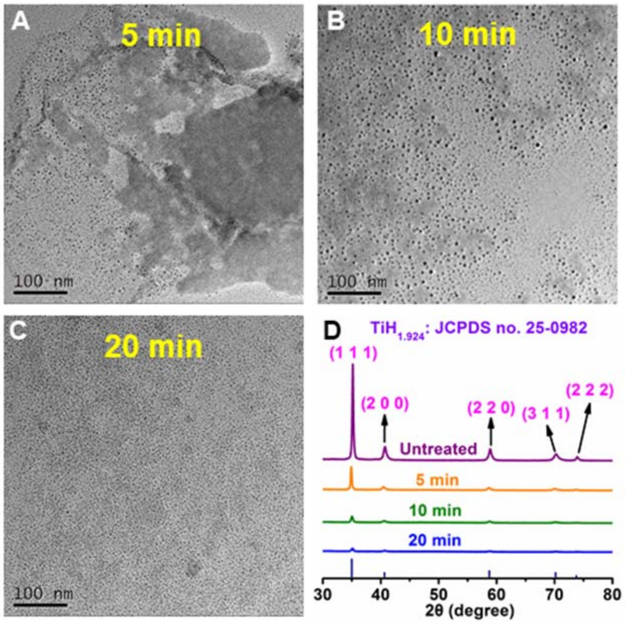

Supplementary Figure 1. (A-D) TEM images (A-C) and XRD spectra (D) of $TiH_{1.924}$ nanodots produced by liquid-phase exfoliation in NMP after sonication for various periods of time (5, 10, and 20 min). A representative image of three biological replicates from each group is shown in (A-C).

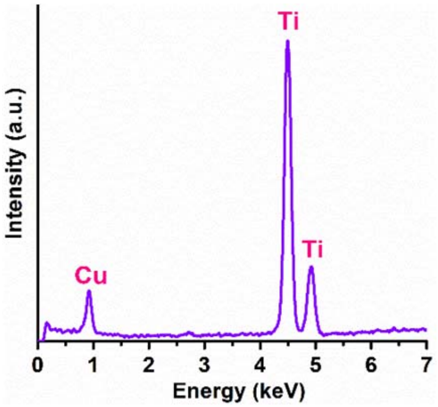

Supplementary Figure 2. EDS spectrum of as-made $TiH_{1.924}$ nanodots.

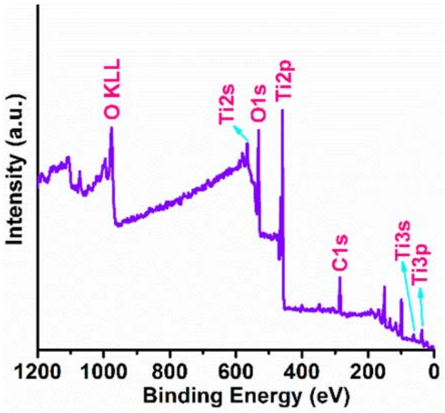

Supplementary Figure 3. The full XPS spectrum of $TiH_{1.924}$ nanodots.

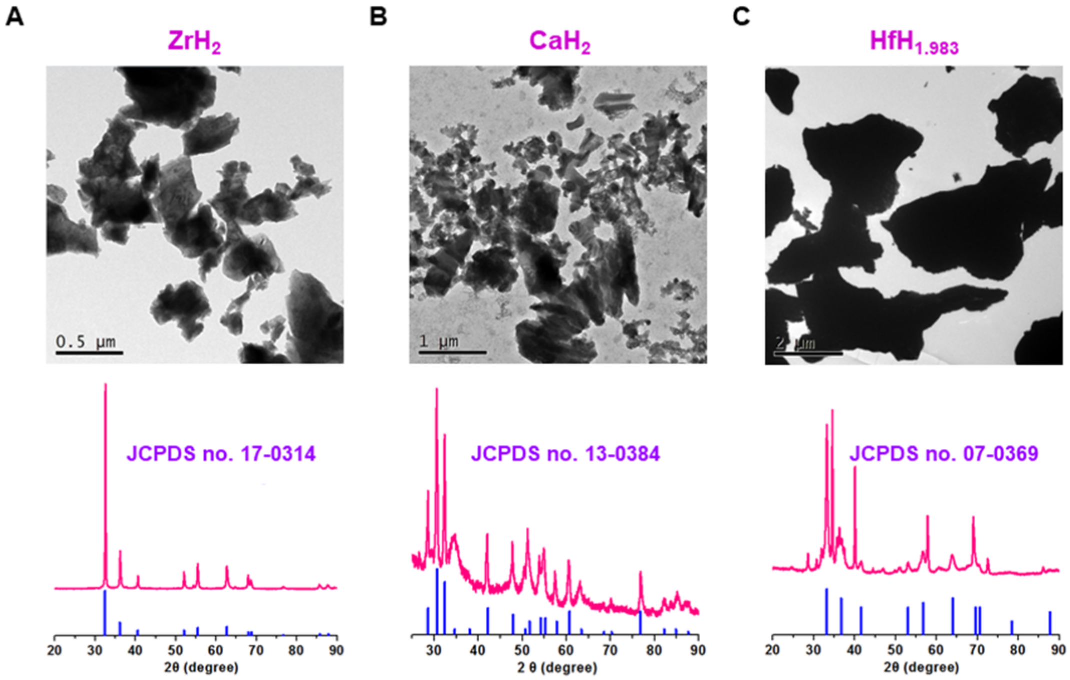

Supplementary Figure 4. TEM images and XRD spectra of the commercial ZrH_2 (A), CaH_2 (B), and $HfH_{1.983}$ (C) powders. A representative image of three biological replicates from each group is shown in (A-C).

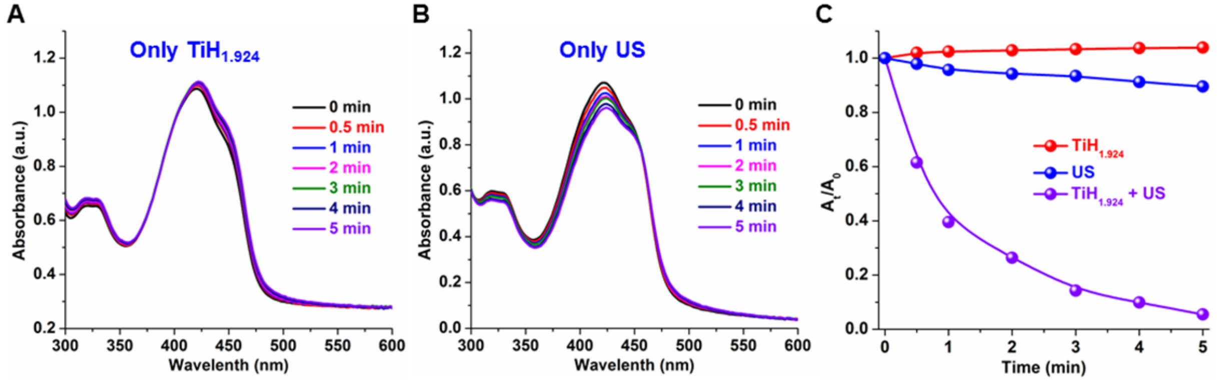

Supplementary Figure 5. (A&B) Time-dependent oxidation of DPBF by $TiH_{1.924}$ along (A) and the US along (B). (C) Comparison of DPBF oxidation by $TiH_{1.924}$ only, US only, and $TiH_{1.924}$ plus US treatment.

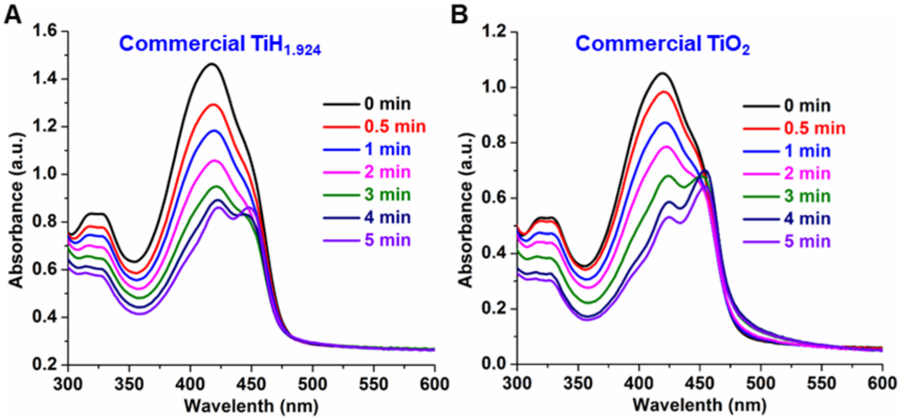

Supplementary Figure 6. Time-dependent oxidation of DPBF by US-activated commercial $TiH_{1.924}$ (A) and TiO_2 (B).

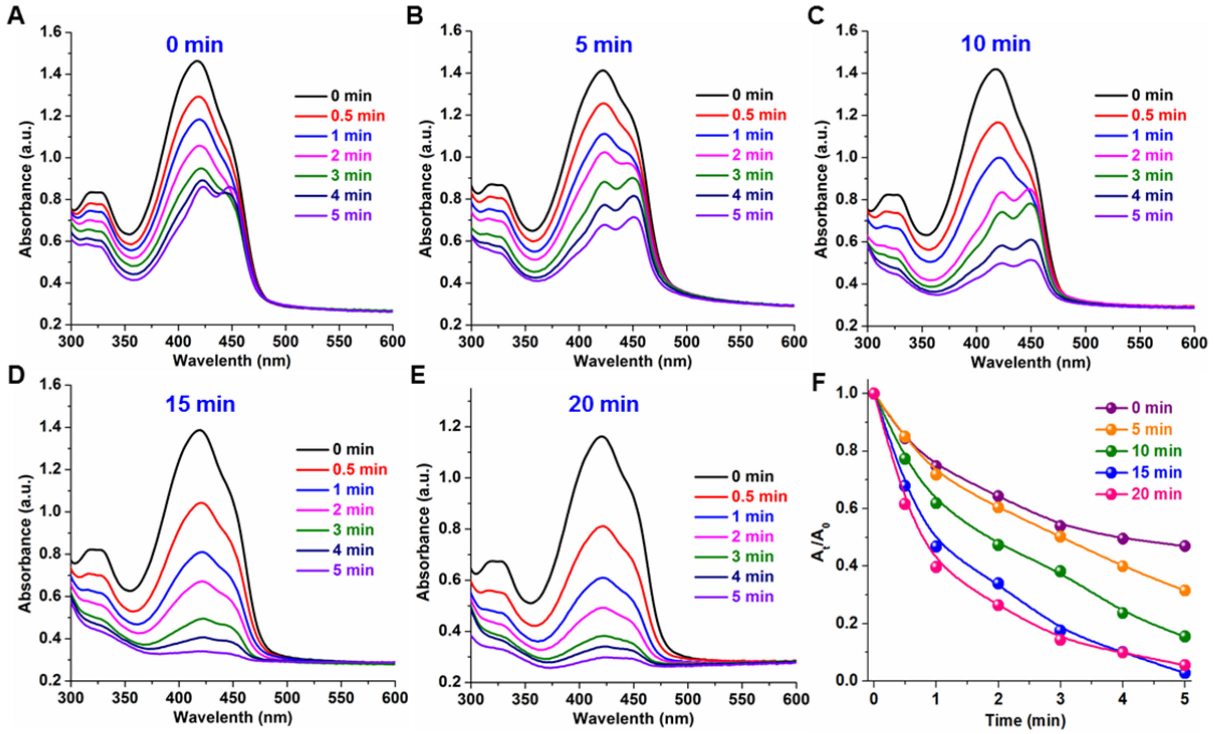

Supplementary Figure 7. (A-E) UV-VIS spectra to time-dependent oxidation of DPBF by US-activated $TiH_{1.924}$ with the different exfoliated degrees (exfoliated time: 0, 5, 10, 15, and 20 min). (F) Comparison of DPBF oxidation by $TiH_{1.924}$ produced by various exfoliated time periods.

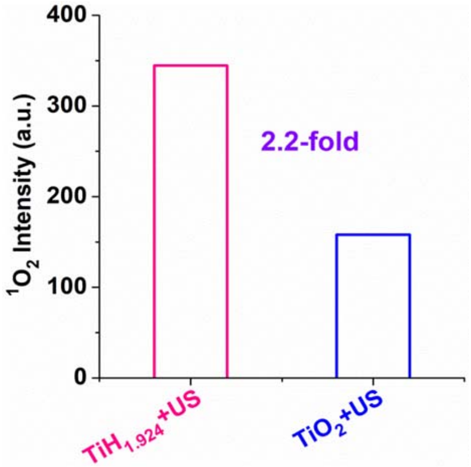

Supplementary Figure 8. Quantitative analysis of $^{1}O_2$ generation for the two groups based on data in Figure 3D.

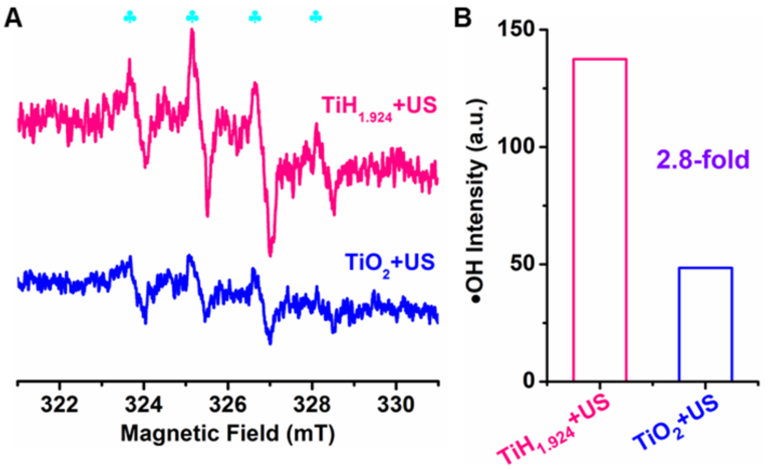

Supplementary Figure 9. (A) ESR spectra demonstrating ROS (•OH) generation for $TiH_{1.924}$ and TiO_2 under US irradiation for 1 min. (B) Quantitative analysis of •OH generation for these two groups as indicated.

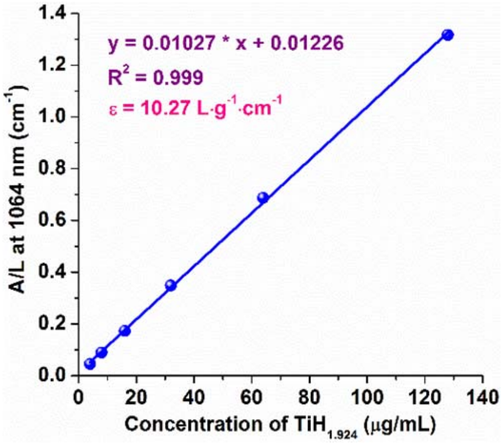

Supplementary Figure 10. Mass extinction coefficient of $TiH_{1.924}$ nanodots at 1064 nm (NIR-II).

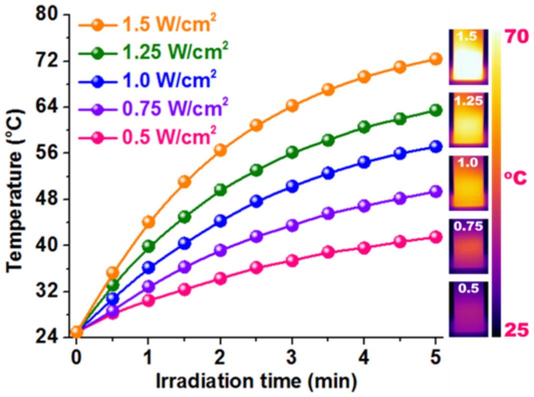

Supplementary Figure 11. Laser power-dependent photothermal heating curves of $TiH_{1.924}$ nanodots (0.5, 0.75, 1.0, 1.25, and 1.5 W•cm^{-2}).

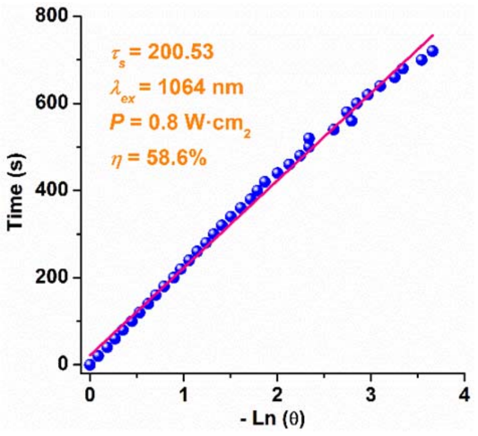

Supplementary Figure 12. Time constant (τ_s) for the heat transfer from the system determined by applying the linear time data from the cooling period.

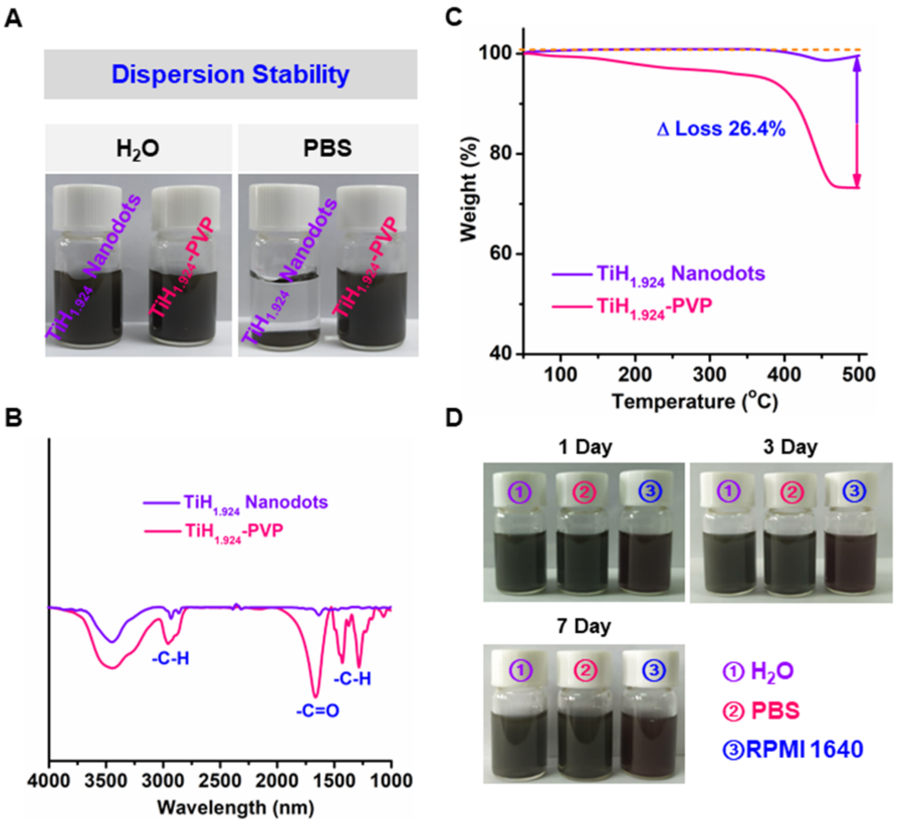

Supplementary Figure 13. (A) The photographs of $TiH_{1.924}$ and $TiH_{1.924}$-PVP dispersed in the H_2O and PBS. (B) Fourier transforms infrared spectrometry (FTIR) spectra of $TiH_{1.924}$ and $TiH_{1.924}$-PVP samples. (C) Thermogravimetric analysis (TGA) of the obtained $TiH_{1.924}$ before and after surface modification. (D) The photographs of $TiH_{1.924}$-PVP in different buffers including H_2O, PBS, and RPMI 1640 cell culture medium for 1, 3, and 7 days.

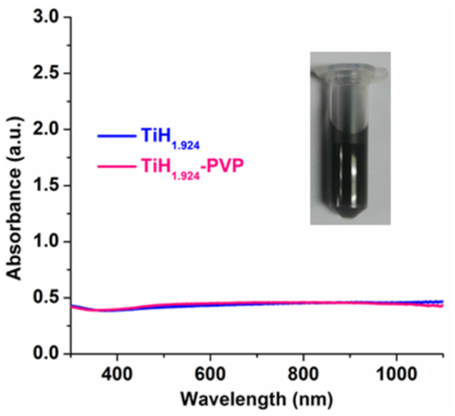

Supplementary Figure 14. UV-vis-NIR absorbance spectra of $TiH_{1.924}$ nanodots before and after PVP modification. Insert is the photograph of $TiH_{1.924}$-PVP. The insert is the photograph of $TiH_{1.924}$-PVP solution.

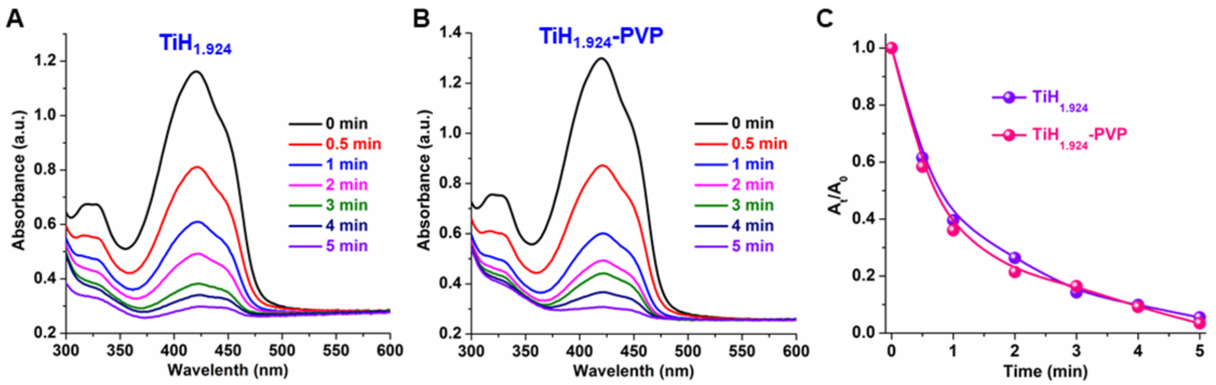

Supplementary Figure 15. (A&B) Time-dependent oxidation of DPBF by US-activated $TiH_{1.924}$ (A) and $TiH_{1.924}$-PVP (B). (C) Comparison of DPBF oxidation by $TiH_{1.924}$ and $TiH_{1.924}$-PVP under US irradiation for 5 min.

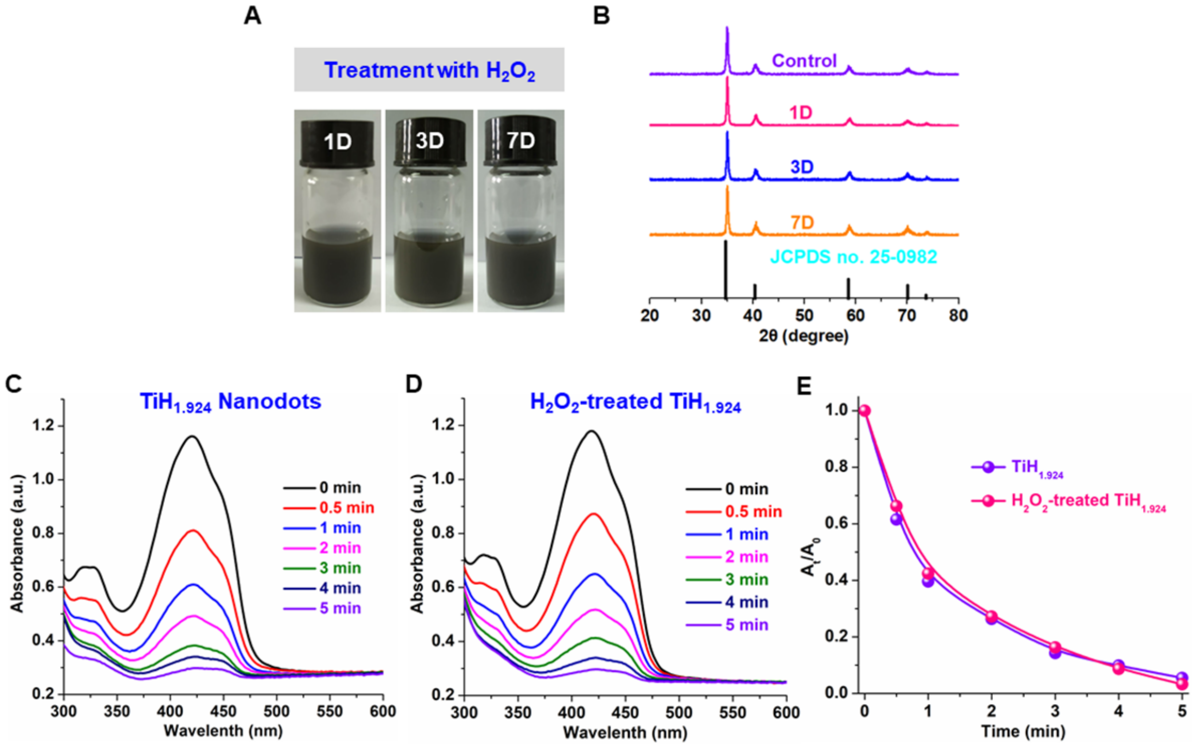

Supplementary Figure 16. (A&B) The photographs (A) and XRD spectra (B) of $TiH_{1.924}$ nanodots after treated with H_2O_2 (1 mM) for 7 days. (C&D) Time-dependent oxidation of DPBF by US-activated $TiH_{1.924}$ nanodots (C) and H_2O_2-treated $TiH_{1.924}$ (D). (E) Comparison of DPBF oxidation by untreated $TiH_{1.924}$ and H_2O_2-treated $TiH_{1.924}$ under US irradiation.

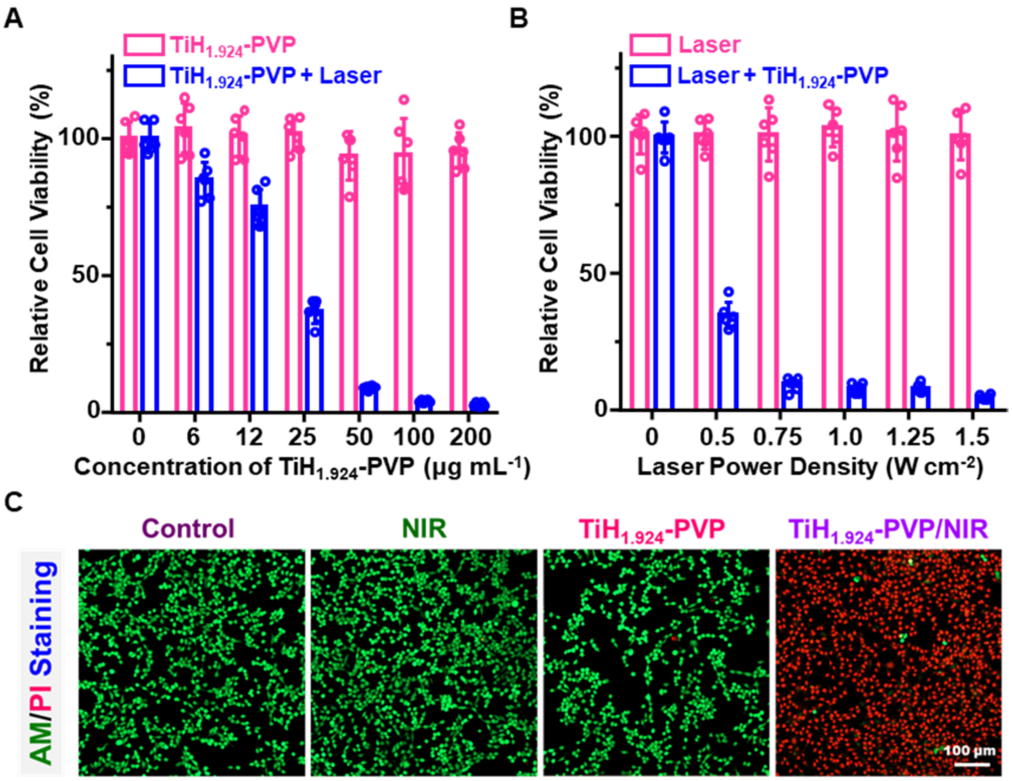

Supplementary Figure 17. (A) The relative viabilities of 4T1 cells after incubation with different concentrations of $TiH_{1.924}$-PVP in the presence or absence of laser irradiation (n=6 biologically independent samples). (B) The relative viabilities of 4T1 cells after PTT with $TiH_{1.924}$-PVP for varied laser irradiation durations (n=6 biologically independent samples). (C) Confocal images of 4T1 cells stained with Calcein AM (green, live cells) and propidium iodide (red, dead cells) after different treatments ($TiH_{1.924}$-PVP: 50 μg•mL^{-1}, NIR laser: 1064 nm, 0.8 W•cm^{-2}, 10 min). Error bars= standard deviation (n=6). Data are presented as mean values ±SD. A representative image of three biological replicates from each group is shown.

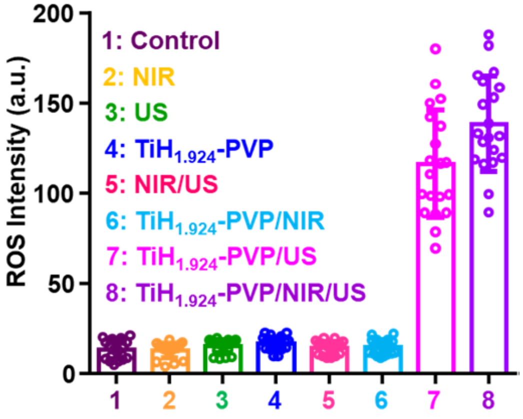

Supplementary Figure 18. Quantitative analysis of intracellular ROS generation for cells in different groups as indicated based on confocal fluorescence images in Figure 4E (n=20 cells examined over independent micrographs). Data are presented as mean values ±SD.

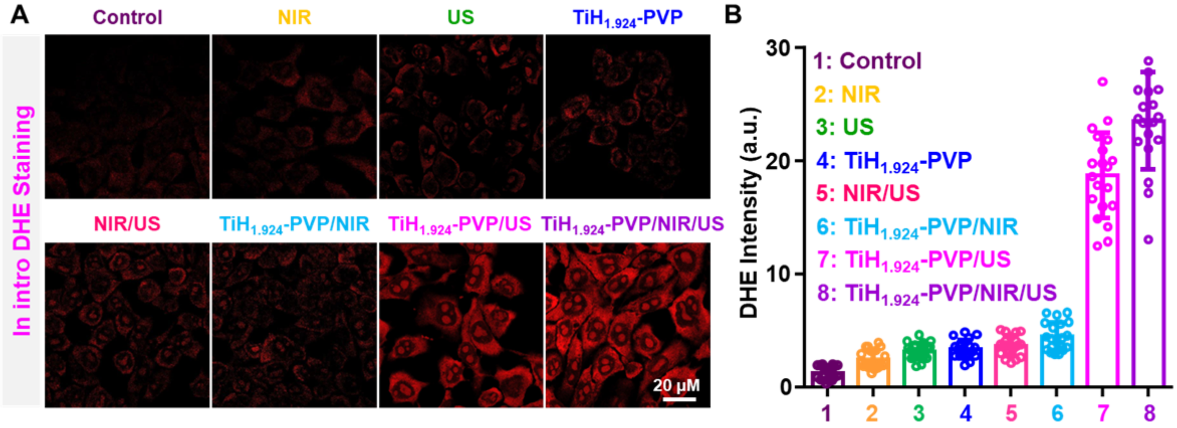

Supplementary Figure 19. (A) Confocal images of 4T1 cells stained with DHE probe after various treatments. A representative image of three biological replicates from each group is shown. (B) Quantitative analysis of intracellular red fluorescent signals in different groups as indicated (n=20 cells examined over independent micrographs). Data are presented as mean values ±SD.

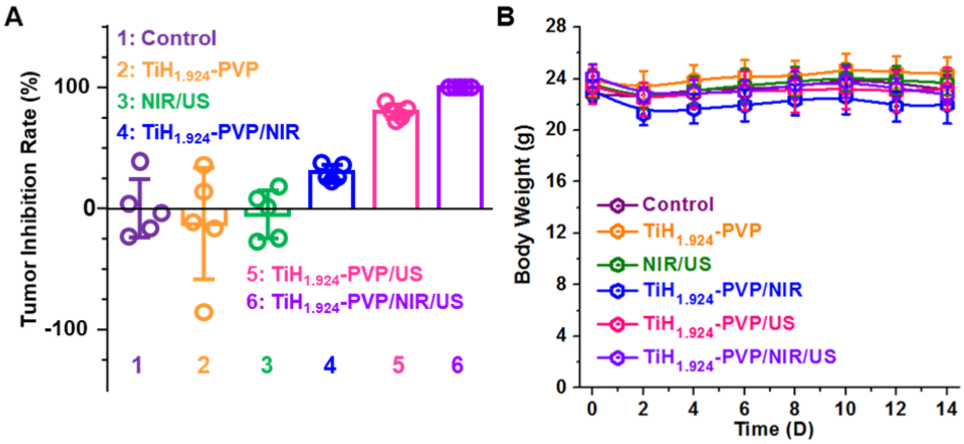

Supplementary Figure 20. (A) Tumor inhibition rates of different treated groups. (B) The body weight variation of mice after various treatments (n=5 biologically independent mice). Data are presented as mean values ±SD.

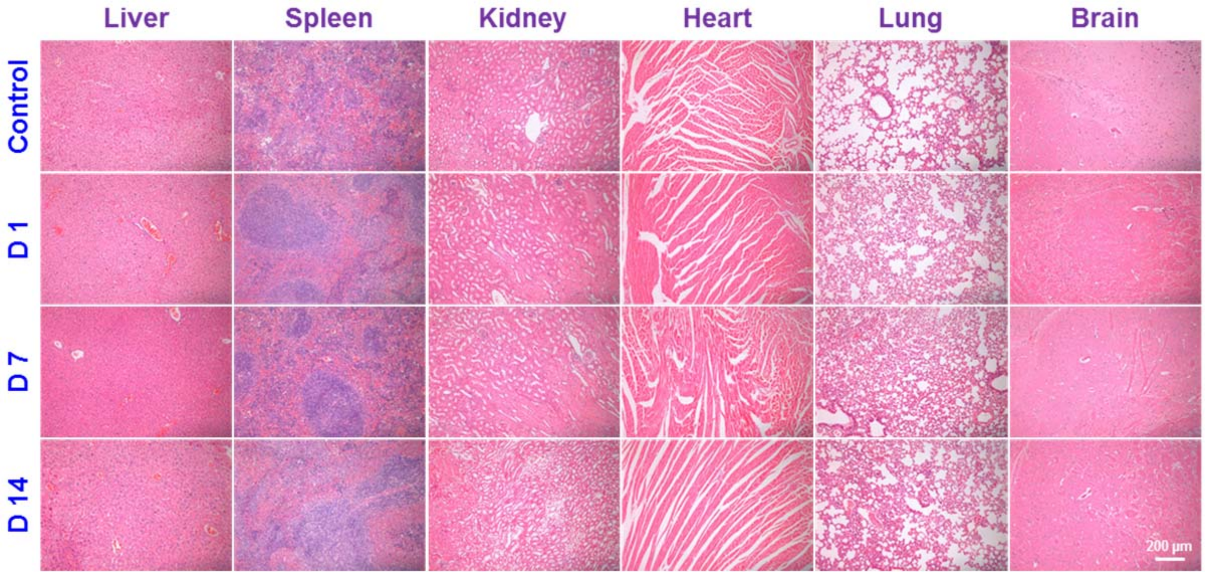

Supplementary Figure 21. H&E staining of major organs (liver, spleen, kidney, heart, lung, and brain) to examine their histological changes after $TiH_{1.924}$-PVP treatment at 1, 7, and 14 days p.i. A representative image of three biological replicates from each group is shown.
