# Supplementary material for: Preparation of TiH1.924 nanodots by liquid-phase exfoliation for enhanced sonodynamic cancer therapy
Source: Nat Commun. 2020 Jul 24;11:3712. doi: 10.1038/s41467-020-17485-x (PMC7381661; doi:10.1038/s41467-020-17485-x)
Supplement: Supplementary file 3 — Reporting Summary [file 41467_2020_17485_MOESM3_ESM.pdf]

nature research

Corresponding author(s): Zhuang Liu

Last updated by author(s): ~~Apr 24, 2020~~

Reporting Summary

Nature Research wishes to improve the reproducibility of the work that we publish. This form provides structure for consistency and transparency in reporting. For further information on Nature Research policies, see Authors & Referees and the Editorial Policy Checklist .

Statistics

For all statistical analyses, confirm that the following items are present in the figure legend, table legend, main text, or Methods section.

n/a	Confirmed	
☐	☒	The exact sample size (*n*) for each experimental group/condition, given as a discrete number and unit of measurement
☐	☒	A statement on whether measurements were taken from distinct samples or whether the same sample was measured repeatedly
☐	☒	The statistical test(s) used AND whether they are one- or two-sided<br>*Only common tests should be described solely by name; describe more complex techniques in the Methods section.*
☐	☒	A description of all covariates tested
☐	☒	A description of any assumptions or corrections, such as tests of normality and adjustment for multiple comparisons
☐	☒	A full description of the statistical parameters including central tendency (e.g. means) or other basic estimates (e.g. regression coefficient) AND variation (e.g. standard deviation) or associated estimates of uncertainty (e.g. confidence intervals)
☐	☒	For null hypothesis testing, the test statistic (e.g. *F*, *t*, *r*) with confidence intervals, effect sizes, degrees of freedom and *P* value noted<br>*Give P values as exact values whenever suitable.*
☒	☐	For Bayesian analysis, information on the choice of priors and Markov chain Monte Carlo settings
☒	☐	For hierarchical and complex designs, identification of the appropriate level for tests and full reporting of outcomes
☒	☐	Estimates of effect sizes (e.g. Cohen's *d*, Pearson's *r*), indicating how they were calculated

Our web collection on statistics for biologists contains articles on many of the points above.

Software and code

Policy information about availability of computer code

Data collection	TEM, XPS, XRD, ESR, UV-vis, Infrared Camera, Confocal Microscopy, PA Tomography, ChemDraw, Excel, Origin, ImageJ, LAS AF Lite
Data analysis	All statistical analyses were performed on Origin 8.5 and Graphpad Prism.

For manuscripts utilizing custom algorithms or software that are central to the research but not yet described in published literature, software must be made available to editors/reviewers. We strongly encourage code deposition in a community repository (e.g. GitHub). See the Nature Research guidelines for submitting code & software for further information.

Data

Policy information about availability of data

All manuscripts must include a data availability statement. This statement should provide the following information, where applicable:
- Accession codes, unique identifiers, or web links for publicly available datasets
- A list of figures that have associated raw data
- A description of any restrictions on data availability

We have provided a full data availability statement in the manuscript.

Field-specific reporting

Please select the one below that is the best fit for your research. If you are not sure, read the appropriate sections before making your selection.

☒ Life sciences ☐ Behavioural & social sciences ☐ Ecological, evolutionary & environmental sciences

For a reference copy of the document with all sections, see nature.com/documents/nr-reporting-summary-flat.pdf

Life sciences study design

All studies must disclose on these points even when the disclosure is negative.

Sample size	Sample sizes were determined according to previous experimental experience.
Data exclusions	No data were excluded.
Replication	Experiments were repeated and experimental findings were reproducible.
Randomization	Mice were allocated randomly to each treatment group.
Blinding	Mice were used in this work. Blinding and randomization were applied to all experiments.

Reporting for specific materials, systems and methods

We require information from authors about some types of materials, experimental systems and methods used in many studies. Here, indicate whether each material, system or method listed is relevant to your study. If you are not sure if a list item applies to your research, read the appropriate section before selecting a response.

Materials & experimental systems

n/a	Involved in the study	
☐	☒	Antibodies
☐	☒	Eukaryotic cell lines
☒	☐	Palaeontology
☐	☒	Animals and other organisms
☒	☐	Human research participants
☒	☐	Clinical data

Methods

n/a	Involved in the study	
☒	☐	ChIP-seq
☒	☐	Flow cytometry
☒	☐	MRI-based neuroimaging

Antibodies

Antibodies used	Anti-pimonidazole mouse monoclonal antibody conjugated to FITC (FITC-Mab1, Hypoxyprobe Inc.) and Alex 488-conjugated goat anti-mouse secondary antibody (Jackson Inc.) for hypoxia staining. Rat anti-CD31 mouse monoclonal antibody (Jackson Inc.) and Rhodamine-conjugated donkey anti-rat secondary antibody (Invitrogen Inc.) for blood vessel staining.
Validation	All antibodies were verified by the supplier and each lot has been quality tested.

Eukaryotic cell lines

Policy information about cell lines

Cell line source(s)	4T1 breast cancer cells were obtained from American type culture collection (ATCC).
Authentication	The cell line we used was morphologically confirmed according to the information provided by ATCC.
Mycoplasma contamination	All cell lines were tested for mycoplasma contamination. No mycoplasma contamination was found.
Commonly misidentified lines (See ICLAC register)	No commonly misidentified cell lines were used.

Animals and other organisms

Policy information about studies involving animals; ARRIVE guidelines recommended for reporting animal research

Laboratory animals	Six-week-old Balb/c mice (18 ± 2g) were used as animal model in this work.
Wild animals	The study did not involve wild animals.
Field-collected samples	The study did not involve samples collected from field.
Ethics oversight	All animal experiments were carried out under the permission by Soochow University Laboratory Animal Center.

Note that full information on the approval of the study protocol must also be provided in the manuscript.
